# The Child of Promise, Etc.

**Published:** 1882-02

**Authors:** 


THE CHILD OF PROMISE, OR THE ISAAC OF MEDICINE AND ISHMAEL, THE HALF-BROTHER, etc. By *W. M. Cate, M. D.*, etc. H. B. Burnham, Washington, D. C.

In this little book, Dr. Cate gives a brief and interesting sketch of medicine up to Hahnemann's time. He then speaks of Hahnemann's investigations, discoveries and tells of the disgraceful manner in which allopathy—aptly called Ishmael—persecuted Homœopathy.

The book is novel in the way it treats the subject, and the story is interestingly told. We hope it may have wide circulation among the laity, who have too long heard only one side of medical history.

